# Effect of Contact Lens Design on Objective Visual Acuity-Based Parameters in Pre-Presbyopic Patients in Photopic and Mesopic Lighting Conditions

**DOI:** 10.3390/vision7020046

**Published:** 2023-06-18

**Authors:** Orit Sztrigler-Cohen, Nogah Bromberger, Yonina Thee, Rivkah Lender, Hadas Ben-Eli

**Affiliations:** 1 Department of Optometry and Vision Science, Hadassah Academic College, Jerusalem 91010, Israel; 2 Department of Ophthalmology, Hadassah Hebrew University Medical Center, Jerusalem 91120, Israel

**Keywords:** contact lens, spheric, aspheric, pre-presbyopia, mesopic lighting, photopic lighting

## Abstract

Presbyopia is often corrected by progressive soft contact lenses (CL), and the resulting visual acuity-based parameters can be affected by the lens design and pupil size under different lighting conditions. In this study, we examined the effect of CL design (spheric vs. aspheric) on objective parameters of visual acuity-based parameters under mesopic vs. photopic lighting conditions. In a prospective, double-blind study, pre-presbyopic and presbyopic patients were fitted with spheric (Dispo Silk; 8.6 base curve, 14.2 diameter) and aspheric (Dispo Aspheric; 8.4 base curve, 14.4 diameter) CLs. The low contrast (10%) and high contrast (100%) visual acuity (VA), amplitude of accommodation (AA) (push-away method, Diopters) and distance contrast sensitivity (CS) (FACT chart, cycles per degree (CPD)) were measured with both types of CLs under mesopic and photopic lighting conditions. The eye with the better visual acuity was tested and analyzed. Thirteen patients (age range: 38–45 years) were included. The mean CS was significantly better with spheric compared to aspheric lenses for low spatial frequencies (3 CPD: 81.69 ± 7.86, 67.62 ± 5.67, respectively; *p* < 0.05), though there was no significant difference for lower or higher spatial frequencies (1.5, 6, 12, 18 CPD). The low-contrast (10%) and high-contrast (100%) VAs were not different between the two lens designs. However, there were significant differences between near VA, distance low-contrast VA and AA obtained under mesopic (dim) vs. photopic (bright) conditions with the aspheric design correction modality. In conclusion, photopic lighting conditions improved both the visual acuity and measured amplitude of accommodation with both lens designs, though the amplitude of accommodation was significantly higher with aspheric lenses. However, contrast sensitivity demonstrated the superiority of the spheric lens at a 3 CPD spatial frequency. This suggests that the ideal lens differs from patient to patient, depending on the visual demands.

## 1. Introduction

Presbyopia is a physiological condition that develops with age, around the fifth decade of life, and manifests in an irreversible decrease in the amplitude of accommodation. This causes blurring and discomfort when working at near [1]. When these visual symptoms occur between the ages of 35 and 39, it is referred to as pre-presbyopia [2]. With the global population of persons aged 65 years and older projected to double to 1.5 billion by 2050, constituting 16% of the world population, presbyopia may become one of the most important visual concerns [3]. In developed countries, where both personal and professional daily life often involve extensive reading and screen time, the visual demands for near and intermediate vision are essential for the young population and presbyopes alike.

The optical correction possibilities of presbyopia include reading glasses, multifocal glasses with an addition of plus power in the lower portion of the lens and several different contact lens (CL) designs [4]. CLs for presbyopia correction can be grouped into three main categories: supplemental spectacle correction over contact lenses, monovision with one eye corrected for near and the other for distance and simultaneous vision multifocal/progressive contact lenses [5]. Simultaneous vision is achieved when distance and near powers are positioned within the pupillary area at the same time, for all gaze positions [6]. To enable this, progressive CLs are designed with concentric rings of alternating optical powers, thereby providing a solution for distance and near vision [7]. This design provides an elegant solution for near and distance vision, requiring only one type of optical correction and allowing for full binocular vision, but the primary disadvantages of this optical correction are the high cost, reduced contrast sensitivity and, for some, a difficult adaptation process. However, a 2016 study demonstrated that 91% of CL wearers aged 35–55 years prefer to continuing to wear CLs even when there are visual symptoms of presbyopia [8]. It is therefore of great importance to provide optimal and affordable CL correction for presbyopic patients.

Contact lenses with an aspheric design may provide a good option for patients with pre-presbyopia and early presbyopia. These lenses are built with a gradual change in the lens curvature on the front or the back surface. The progressive flattening in an aspheric lens is greater than that in a spheric lens, thus creating an increase in plus power from the center to the circumference of the lens [9]. In this way, an aspheric lens essentially simulates a multifocal lens with the addition of a low plus power for near [10]. Progress in the field of metalenses may revolutionize the traditional multifocal contact lens design and contribute to advancements in optical technologies. These advancements could include applications in ophthalmology for studying corneal cells and understanding the behavior of contact lens materials and may impact the development of contact lenses with enhanced functionalities or improvements in manufacturing processes. Additionally, these advancements will further the study of fluid dynamics, including the study of tear film and contact lens interactions. However, currently, the available contact lens solutions for presbyopes include multifocal lenses and, within a limited capacity, aspheric lenses [11,12,13,14].

Because CL correction for presbyopia is based on changes in optical power within the pupillary area, visual acuity (VA) with progressive CLs is highly affected by the size of the pupil and lens design [15]. A small pupil with a center-near lens, for example, where the optical power at the center of the contact lens corrects for near vision, may cause significant symptoms for distance vision. Similarly, a large pupil may cause photopic phenomena with a CL designed with a power profile favoring near vision over a large area of its geometry [15]. Changes in pupil size are an integral part of the visual system that control the amount of light entering the eye and contribute to the image focus on the retina. The pupil constricts under photopic lighting conditions, dilates under mesopic conditions and constricts with the activation of accommodation [16]. While presbyopes have decreased accommodative power, pupil constriction still occurs when working at near, albeit to a lesser extent [17]. It is therefore crucial to measure the pupil diameter when fitting a contact lens for the correction of presbyopia or pre-presbyopia. 

Pupil size is an important factor for vision in multifocal contact lenses. An association between the pupil size and the power distribution of the multifocal contact lens was previously reported, emphasizing the need for the accurate evaluation of the optical properties of a multifocal CL, as well as the patient’s visual needs for achieving the optimal visual outcome [18]. Moreover, a large pupil size and the multifocal CL design may induce light distortion effects [19] and also affect the distance and near VA [20], so a small pupil is associated with lower near VA [21]. The main factors affecting the pupil size are the luminance, refractive error and age. The pupil diameter becomes smaller with increasing age, with a significant difference between the pre-presbyopes and established presbyopes, and the this effect is most marked at low luminance [22].

The effect of the CL design on presbyopic patients has been described in several studies [23,24,25]. However, these studies did not include pre-presbyopic patients and did not cover the current set of functional visual exams in relation to different contact lens surface designs. In this study, we aimed to compare the objective parameters of visual acuity-based parameters in pre-presbyopes and early presbyopes with spheric vs. aspheric CL designs and to assess this effect under mesopic vs. photopic lighting conditions with different pupil sizes.

## 2. Materials and Methods

### 2.1. Participants

This study included healthy patients between the ages of 38 and 45, i.e., pre- and early presbyopes, with amplitudes of accommodation (AA) ranging between 3.00 and 5.50 diopters (D), as measured using the push-away test [26]. The patients all underwent a full refractive examination and fitting, regardless of whether or not they had worn contact lenses in the past. Patients with active ocular pathologies, corneal diseases or eye dryness and those who had undergone any corneal or cataract surgery were excluded. Additionally, diabetic patients, pregnant women and patients that refused to sign an informed consent form were not included in the study. 

The participants were myopic up to −6.00 D, with astigmatism up to −0.75 D and with VA values of at least 0.7 decimals (dec) in the tested eye. Hyperopes were excluded. Monocular visual parameters were measured in order to avoid confounding effects of binocular summation [27]. Only the eye with the best acuity and the lowest astigmatism of each participant was tested and included in the analysis in order to avoid artificially strengthening the statistical significance due to the strong correlations in values from the two eyes [28]. In cases where the eye with the better acuity also had the higher amount of astigmatism, the eye with the lower astigmatism was included. Patients were recruited in the CL clinic of the Optometry Department at Hadassah Academic College as well as via advertisements in social networks. Each participant received an oral and written explanation of the study aims and methods and signed an informed consent form. Data were coded and analyzed anonymously.

### 2.2. Contact Lens Parameters

Each patient was fitted with a spheric and aspheric hydrogel lens. The spheric lenses were Dispo Silk (CooperVision-Soflex, Bar Lev, Israel, Ltd.) daily lenses made of Vifilcon with a water content of 59.0%, oxygen transmissibility (DK/L) of 20 × 10^−9^ @ −3.00 DS, center thickness of 0.08 mm @ −3.00 DS, base curve of 8.6 mm and diameter of 14.2 mm. The aspheric lenses were Dispo Aspheric (CooperVision-Soflex, Bar Lev, Israel, Ltd.) monthly lenses made of Methafilcon A with a water content of 55.0%, DK/L of 27 × 10^−9^ @ −3.00 DS, center thickness of 0.07 mm @ −3.00 DS, edge thickness of 0.07 mm, base curve of 8.4 mm and diameter of 14.4 mm.

### 2.3. Data Collection and Study Procedure

All study tests were carried out in a prospective, double-blind manner and in the same examination room to ensure uniform conditions of the lighting and equipment. Testing was performed in the CL clinics at Hadassah Academic College. 

The main outcome measures were high-contrast (100%) and low-contrast (10%) distance VA (Snellen chart, decimal units), near VA (Rosenbaum Pocket Vision Screener (RPVS), Jaeger (J) units), CS (FACT chart, cycles per degree (CPD)) (StereoOptical, Chicago, IL, USA) and AA (push-away method). Distance VA was measured at 3 m with an appropriately calibrated projector, and near VA was measured at 33 cm. CS was measured at 3 m.

Each participant underwent entrance tests that included a full refractive exam, an eye health test, the tear break up time (TBUT), a measurement of eye lid tension and vertical palpebral fissure height and a slit lamp examination of the cornea and conjunctiva (CSO 2015, Scandicci, Italy). Monocular and binocular VA were measured for distance with high- (100%) and low (10%)-contrast projectors and for near with the RPVS.

CL fitting tests were carried out using a double-blind crossover procedure. Monthly spheric and aspheric CLs were assigned randomly so that half of the participants received the spherical lens design first and the aspheric lens second, while the other half began the study with the aspheric design. The CL fitting was carried out by an optometrist (NB) using a uniform protocol and included criteria of limbus to limbus (≥1 mm), lag and sag (0.5–1 mm), a displacement test and blink movement. After 15 min with the CL in the eye, the participants were tested again to examine the distance and near VA and contrast sensitivity, and AA was measured with the optimal correction over the CL under maximum lighting conditions by a second examiner (Y.T). 

The lighting in the room was dimmed to a pre-set minimum that ensured a change of at least 2 mm in the pupil size between the maximum and minimum illumination for each participant, based on the norms for pupil size changes in the study population age group [7]. Pupil size was measured under mesopic conditions (35 lx) and photopic conditions (108 lx) with a Gossen 6.73-422 PANLUX electronic luxmeter in order to ensure that the change in the pupil size was as expected. The VA, contrast sensitivity and amplitude of accommodation were measured again by a third examiner (O.C).

After a wash-out period of 30 min, the patient was fitted with the second lens design. This, too, was left in the eye for 15 min, after which all the above tests were repeated under the two lighting conditions.

### 2.4. Power Calculation

A sample size of 13 patients with an expected change of a two-line difference between low- and high-contrast VA [29], with α = 0.05, would yield a power of only 30%. However, a change of 2 mm in the pupil size between mesopic and photopic lighting may induce a 1.40 diopter change (0.7 for 1 mm) [7], which is expected to change the visual acuity by three to four lines [30] in VA between the lighting conditions, with α = 0.05 yielding a power of 80% for this sample size. An additional calculation based on the actual difference of one line in the visual acuity (decimal acuity) between two multifocal lenses, as used by Bakaraju et al., yielded an effect size of 0.64. A two-tailed test with a 5% level of significance and a sample size of 13 participants yielded a power of 78% (Calculated by G*Power software, version 3.1.9.4) [31].

### 2.5. Statistical Analysis

The normality of the data was tested using the Kolmogorov Smirnov test. The comparison of the mean VA, AA and CS between the different lenses and between the different pupil sizes was carried out by the Friedman test for paired samples. Bonferoni correction was used for post hoc analysis and multiple comparisons. A statistically significant result was considered as *p* < 0.05 in a two-tailed test. The analysis was performed using SPSS software (IBM SPSS Statistics, Version 27.0, Chicago, IL, US, Armonk, NY, US: IBM Corp).

## 3. Results

Fifteen participants were recruited for this study, but two of them did not fulfill the inclusion criteria due to a high cylinder (>3.00 D). A total of 13 eyes of 13 participants were included in the analysis. The eye with the better VA and/or the lower cylinder of each participant was included (61.5% right eyes). Of these, 53.8% were females, with an age range of 38–45 years and a mean age of 42.5 ± 2.4 years. The basic characteristics of the study participants are described in Table 1. Individual parameters are described in Appendix A.

**Figure 1:**
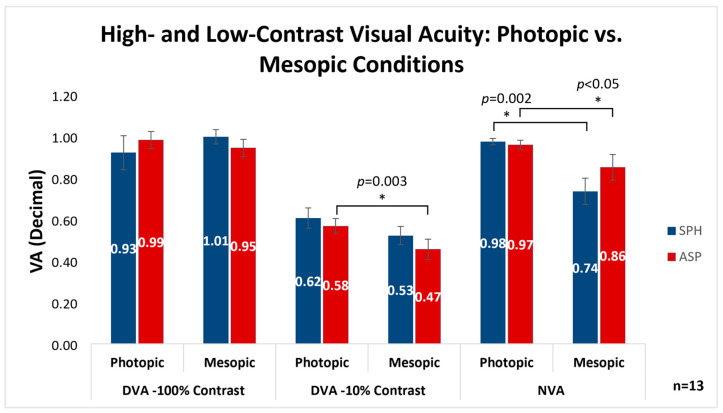
Comparison between the mean distance visual acuity in high and low contrast and the near visual acuity obtained with spheric and aspheric lenses with different pupil sizes induced by photopic and mesopic lighting. The low-contrast distance VA with the aspheric lens was improved under photopic conditions. The near VA was improved with both lens types under photopic conditions. Visual acuity units are decimals. DVA = distance visual acuity; NVA = near visual acuity; SPH = spheric lens; ASP = aspheric lens; CS = contrast sensitivity. Error bars indicate SE. * *p* < 0.05; Friedman test.

Figure 1 demonstrates the comparison between the mean VA ± SE with spheric and aspheric lenses in six conditions: distance VA under photopic and mesopic lighting conditions with 100% and 10% contrast, and near VA under photopic and mesopic lighting conditions. When examining the same lens type under different lighting conditions at a distance, the only statistically significant difference was found in the aspheric lens, which demonstrated improved VA under photopic vs. mesopic lighting with 10% contrast (0.58 ± 0.04, 0.47 ± 0.05, respectively; *p* = 0.003). At near, however, VA was significantly higher under photopic vs. mesopic lighting with both the aspheric (0.97 ± 0.02, 0.86 ± 0.06, respectively; *p* = 0.049) and spheric (0.98 ± 0.02, 0.74 ± 0.06, respectively; *p* = 0.002) lenses. When comparing the two lens designs across the different lighting conditions, distances and contrasts, no statistically significant differences were found.

**Figure 2:**
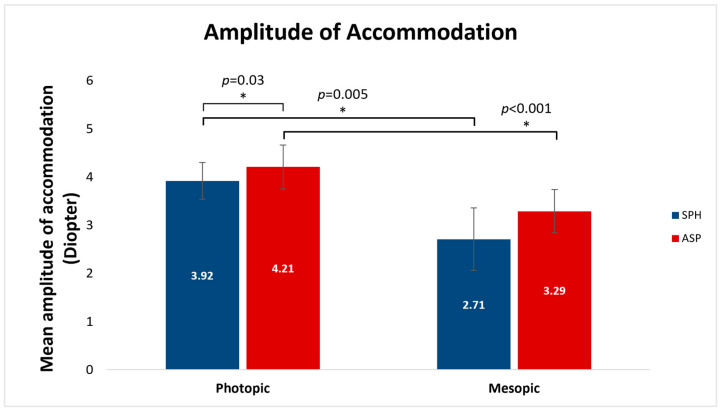
Comparison of the mean amplitudes of accommodation (Diopter) obtained with spheric and aspheric contact lenses obtained in photopic vs. mesopic lighting. The measured amplitude of accommodation was increased under photopic conditions with both spheric and aspheric lens designs. Under photopic conditions, the aspheric lens design yielded a higher amplitude of accommodation. Error bars indicate SE. * *p* < 0.05; Friedman test.

Figure 2 shows the comparison between the mean measured amplitudes of accommodation in diopters (D) ± SE obtained with the two lenses with different pupil sizes induced by different lighting conditions. AA was significantly higher under photopic vs. mesopic conditions with both the spheric (3.92 ± 0.37 D, 2.71 ± 0.65 D, respectively; *p* = 0.005) and the aspheric (4.21 ± 0.45 D, 3.29 ± 0.45 D, respectively; *p* < 0.001) lens designs. Additionally, under photopic lighting conditions, the mean AA in aspheric lenses was significantly higher than the AA in spheric lenses (4.21 ± 0.45 D, 3.92 ± 0.37 D, respectively; *p* = 0.03). Under mesopic lighting, no statistically significant differences were found in the mean AA between the spheric and the aspheric lenses (*p* > 0.05).

**Figure 3:**
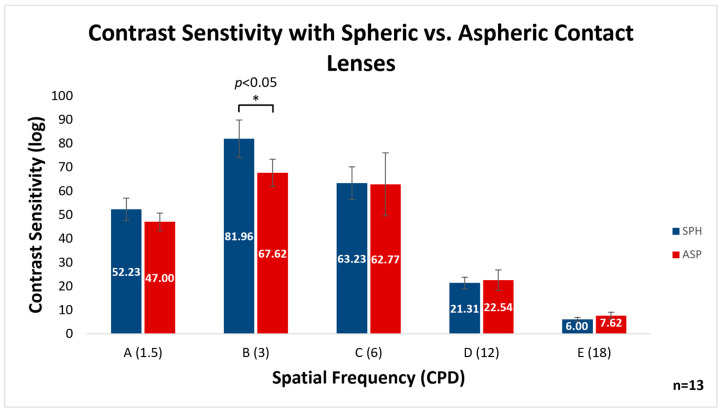
Comparison of the mean contrast sensitivity of different spatial frequencies obtained with spheric and aspheric contact lenses in the FACT chart. Contrast sensitivity was improved with the spheric lens design at the 3 CPD frequency. CS = contrast sensitivity; SPH = spheric contact lens design; ASP = aspheric contact lens design; CPD = cycles per degree. Error bars indicate SE. * *p* < 0.05; Friedman test.

The comparison of CS between the two lens designs across five spatial frequencies is demonstrated in Figure 3. It can be seen that the mean CS ± SE was significantly better with the spheric vs. aspheric lenses at the 3 CPD frequency (column B in the FACT chart) (81.96 ± 7.86, 67.62 ± 5.67, respectively; *p* < 0.05). No statistically significant differences were found for the other spatial frequencies.

## 4. Discussion

This study aimed to compare the objective quality of vision obtained in pre- and early presbyopes with the spheric contact lens compared with the aspheric contact lens designs in a variety of conditions: distance visual acuity with high and low contrast, visual acuity at near, amplitude of accommodation and distance contrast sensitivity.

The main results found in this study were the significant differences in the mean VA under different lighting conditions and contrast levels with the different lenses. The VA with aspheric lenses demonstrated a greater impact of lighting conditions, improving both at distance and near under photopic conditions. Furthermore, while the amplitude of accommodation was similarly improved in both lens types by photopic lighting conditions, the mean amplitude of accommodation was found to be higher with aspheric lenses relative to spheric lenses under photopic conditions. The comparison of contrast sensitivity with different spatial frequencies between the two lens designs demonstrated a significant difference only at 3 CPD, where spheric lenses resulted in a higher CS.

We suggest that any differences in outcome found in the current study were caused by the differences in the front surface design (i.e., spheric vs. aspheric) and not by differences in the physical properties of the lenses. A previous work demonstrated no differences in visual outcomes between monthly and daily lenses with spherical designs [32], and as such, any differences found in this study can be attributed to the front surface curvature.

So far, to our knowledge, no study has been performed comparing VA between spheric and aspheric contact lenses among pre-presbyopes, as well as testing the effect of pupil size on these measures in lenses with different designs. As previously discussed, aspheric lenses have increased plus in the periphery of the optical zone compared to spherical lenses [9]. In this way, an aspheric lens essentially simulates a multifocal lens with the addition of a low plus power for near [10]. For this reason, it was interesting to test visual acuity in mesopic lighting conditions, where the pupil is dilated and a wider portion of the lens surface affects the light entering the pupil. However, no significant difference in near VA under mesopic lighting conditions was observed between the spheric and aspheric lens designs. The significant improvement occurred within lens designs in photopic vs. mesopic lighting conditions, re-enforcing the importance of proper lighting, especially for near work.

It is interesting to note that while no improvement in VA was observed with the use of aspheric contact lenses in the current study, there was a significant increase in the measured amplitude of accommodation. While not an expected finding, this is perhaps more significant in the subjective visual comfort of contact lens users. An increased amplitude of accommodation reduces eye strain and asthenopia and may allow for longer periods of near work without experiencing common symptoms of presbyopia [33]. This increase in the amplitude of accommodation was seen under photopic conditions with both the spheric and aspheric lens designs, though the increased amplitude was significantly higher with the aspheric design.

Photopic conditions trigger pupil constriction, which is essentially a pinhole effect, sharpening the image on the retina and increasing the depth of field and depth of focus [34,35]. An increased depth of field enlarges the range in which the target can be viewed clearly, preventing the need for the reader to use maximal accommodative reserves. Combined with the improved visibility induced by brighter lighting conditions, this translates in objective measurements to a greater amplitude of accommodation. It is important to emphasize that photopic conditions do not affect the eyes’ anatomical functions but rather maximize the accommodative potential.

In the current study, we tested contrast sensitivity at 3 m with the FACT chart and found the results to be superior with the spheric lens design compared to the aspheric one. The improvement was statistically significant only in the measurement of the 3 CPD, probably due to the fact that, at frequencies of 3–6 CPD, the sensitivity to contrast is maximal [36], optimizing the ability to distinguish between more subtle differences. It is possible that these differences are only detectable in frequencies where sensitivity is maximal.

The main limitation of the current study was the small sample size. The sample size provided sufficient power for the study aims when calculated according to expected differences in visual acuity; however, calculation based on an actual difference of one VA line between lenses yielded a medium power level. A larger sample would ensure additional statistically significant results comparing the visual acuity, amplitude of accommodation and contrast sensitivity with the two lens designs and lighting conditions. An additional limitation was the use of decimal units in the analysis of visual acuity results. Because testing was performed using a decimal chart, these units were used for analysis in order to accurately reflect the study procedures. The strengths of the study, however, are the comparisons of multiple parameters under different lighting conditions with the two different lens designs studied within the pre- and early presbyopic age groups. Previous works have compared similar parameters, but there are limited data in the literature regarding different lens surface designs in this age group [23,24,25]. While the findings of this pilot study should be considered preliminary due to the limited sample size, this provides justification for further research of the optical solutions and the resulting visual acuity-based parameters for this population. It is important that future research focus on this demographic, as they still have more accommodative abilities than full presbyopes, yet they present with visual complaints that the younger population does not experience. Including additional subjective and qualitative outcomes such as those described by Jong et al., 2019 and Papas et al., 2009 would further shed light on the visual performance of this study demographic. [37,38]

Considering that the vast majority of contact lens wearers aged 35–55 years, i.e., pre-presbyopes and presbyopes, expressed a preference to continue wearing contact lenses [8], it is important to develop a thorough understanding of the impact of different contact lens designs for this demographic. As the visual system undergoes a change with age, optimal solutions must be offered accordingly. Different lens designs can provide a solution for early presbyopes and eliminate the need to wear multifocal contact lenses, which are a much more costly option. While eyecare providers should always be cognizant of the visual needs of their patients, doing so in this age group is especially impactful and can reduce the visual symptoms of presbyopia.

While our findings do not indicate that one lens is necessarily superior to the other, it can be suggested that patient history and visual needs should be thoroughly investigated and considered when performing a contact lens fitting for pre- and early presbyopes. An aspheric lens, for example, may not be ideal for patients who work both in and out of doors, as the difference in visual acuity under different lighting conditions is more pronounced. However, aspheric lenses were shown to yield a higher mean amplitude of accommodation, which would be ideal for indoor near work, particularly under photopic conditions.

## 5. Conclusions

Photopic lighting conditions improved both the visual acuity and measured amplitude of accommodation with both lens designs, though the amplitude of accommodation was significantly higher with aspheric lenses. However, the contrast sensitivity demonstrated the superiority of the spheric lens at a 3 CPD spatial frequency. This suggests that the ideal lens differs from patient to patient, depending on the visual demands.

## Figures and Tables

**Table 1:** Basic characteristics of the study participants.

Characteristic (N = 13)	Mean ± SD
Mean age (years)	42.5 ± 2.4
Mean SPH (D)	−3.40 ± 1.94
Mean CYL (D)	−0.21 ± 0.27
Male sex N (%)	7 (46.2)
Included eye-RE N (%)	8 (61.5)
Distance BCVA under 100% contrast (Dec)	1.02 ± 0.12
Distance BCVA under 10% contrast (Dec)	0.60 ± 0.13
Near BCVA (Dec)	1.0 ± 0.0
Amplitude of Accommodation (D)	4.38 ± 1.85

SD = Standard Deviation; SPH = Sphere; D = Diopters; CYL = Cylinder; N = Number; RE = Right eye; BCVA = Best Corrected Visual Acuity; Dec = Decimal.

## Data Availability

The study data are available upon request from the corresponding author (H.B.).

## Appendix A

**Table A1:** Participants’ Individual Parameters.

	Sex	Age (Years)	Tested Eye	SPH	CYL	AX	CL Prescription
**1**	F	45	L	−4.25	−0.50	50	−4.25
**2**	F	48	R	−1.75	0.00	0	−1.75
**3**	F	49	R	−5.75	−0.25	173	−5.50
**4**	M	47	L	−2.50	−0.50	83	−2.75
**5**	M	51	L	−1.50	−0.50	90	−1.75
**6**	M	49	R	−5.25	0.00	0	−5.00
**7**	M	49	L	−0.50	0.00	0	−0.50
**8**	M	48	R	−4.75	−0.75	92	−4.75
**9**	M	49	R	−1.75	0.00	0	−1.75
**10**	F	51	L	−4.75	0.00	0	−4.50
**11**	F	44	R	−6.00	0.00	0	−5.50
**12**	F	45	R	−4.50	0.00	0	−4.25
**13**	F	43	R	−1.00	−0.25	9	−1.00
